# Tumor lysis syndrome in children with hematological malignancies: a nephrology perspective in resource-limited settings

**DOI:** 10.3389/fonc.2026.1778776

**Published:** 2026-03-10

**Authors:** Samar Atef Elshafey, Lamiaa Essa, Maha Youssef Zeid, Yasser Wali, Yasmine El Chazli

**Affiliations:** 1 Pediatric Nephrology Unit, Department of Pediatrics, Faculty of Medicine, Alexandria University, Alexandria, Egypt; 2 Borg El-Arab Pediatric Oncology Center, Department of Pediatrics, Faculty of Medicine, Alexandria University, Alexandria, Egypt; 3 Pediatric Hematology/Oncology Unit, Department of Pediatrics, Faculty of Medicine, Alexandria University, Alexandria, Egypt; 4 Department of Child Health, College of Medicine and Health Sciences, Sultan Qaboos University, Muscat, Oman

**Keywords:** acute kidney injury, febuxostat, kidney replacement therapy, phosphate dynamics, rasburicase unvailability, children, tumor lysis syndrome

## Abstract

**Background:**

Tumor lysis syndrome (TLS) is a major metabolic emergency in pediatric oncology and a leading cause of acute kidney injury (AKI) in children with hematological malignancies. Early identification of children at risk for severe AKI remains challenging.

**Methods:**

This retrospective study included 50 children with laboratory or clinical TLS diagnosed according to the Howard–Pui classification. Serial biochemical parameters were analyzed over a 10-day period. AKI severity was classified using the pediatric Risk, Injury, Failure, Loss, End-stage renal disease (pRIFLE) criteria based on changes in estimated glomerular filtration rate (eGFR). Clinical characteristics, biochemical trends (especially phosphate and uric acid), and outcomes were compared between children with mild (pRIFLE 0 [no AKI]/R/I) and severe (pRIFLE-F) AKI.

**Results:**

Twenty-seven patients had acute lymphoblastic leukemia (ALL), and 23 had lymphoma, mainly Burkitt’s lymphoma. Clinical TLS accounted for 86% of cases, and 60% of children developed severe AKI. Severe AKI was significantly associated with spontaneous TLS onset, prolonged TLS duration, increased need for kidney therapy, intensive care admission, and higher mortality. Static demographic characteristics, malignancy type, tumor burden, and radiological findings did not differ between AKI severity groups. While hyperphosphatemia and hyperuricemia were common, dynamic phosphate changes showed the strongest association with AKI severity. The daily rise in serum phosphate before AKI onset demonstrated good discriminatory performance for predicting severe AKI (AUC 0.839), outperforming changes in uric acid.

**Conclusion:**

In pediatric TLS, AKI severity is the main determinant of clinical outcome. Dynamic phosphate kinetics (Delta phosphorus), rather than static biochemical thresholds, represent a robust early biomarker for identifying children at risk of severe AKI and may improve risk stratification, particularly in resource-limited settings.

## Introduction

Tumor Lysis Syndrome (TLS) represents a major metabolic emergency in pediatric oncology, and is most frequently encountered in children with rapidly proliferating hematologic malignancies, particularly ALL. In this population, abrupt cytoreduction following induction chemotherapy, or occurring spontaneously in high-tumor-burden disease, leads to the accelerated release of intracellular potassium, phosphorus, and nucleic acid metabolites into the systemic circulation. The resultant hyperuricemia, hyperphosphatemia, hyperkalemia, and secondary hypocalcemia place children at substantial risk for AKI, cardiac dysrhythmias, and neuromuscular complications (1–3).

The pathomechanism underlying AKI in TLS is complex and entails both crystal-dependent and crystal-independent mechanisms, which can trigger endothelial damage and microvascular dysfunction (3, 4). Crystal deposition in the renal tubules includes uric acid and xanthine crystals or calcium-phosphate due to hyperphosphatemia. Renal blood flow and eGFR were already diminished by up to 50% in cases with mild hyperuricemia, reflecting early kidney involvement (5, 6).

Given the heightened metabolic vulnerability of children and the intensity of the frontline multi-agent chemotherapy regimens, TLS may evolve rapidly and unpredictably. Consequently, meticulous biochemical monitoring and early recognition of dynamic electrolyte and renal parameters are essential for accurate risk stratification, timely prophylaxis, and prevention of progression to severe AKI (1–3).

Reported TLS incidence varies widely, ranging from 3 to 30% of patients with leukemia and non-Hodgkin lymphoma, with spontaneous TLS occurring more frequently in pediatric patients than in adults (7–9). Approximately 20–40% of TLS cases manifest as CTLS. Among children with cancer, TLS–related AKI has been reported in 5 to 40% of cases (8), increasing to as high as 75% in malignancies with a large tumor burden (9). Severe TLS most commonly manifests as kidney injury (7). While reported dialysis rate ranges from 2 to 4% (9), TLS-related mortality remains approximately 1.7% among high-risk patients (10).

Despite advances in TLS prophylaxis and management, risk stratification remains largely based on static biochemical thresholds, which may fail to capture early dynamic changes preceding overt kidney injury (10). Emerging evidence suggests that rapid rises in serum phosphate may serve as an early and sensitive marker of TLS-associated AKI (11, 12). Yet, pediatric data remain limited, particularly in resource-constrained settings where access to rasburicase and kidney replacement therapy (KRT) is restricted.

The estimated incidence rate of childhood cancer in Egypt was 12.1 per 100,000 children (13). Moreover, based on 2020 Global Cancer Statistics (GLOBOCAN) data by the International Agency for Research on Cancer (IARC), Egypt had the greatest number of annual childhood cancer deaths in the Eastern Mediterranean region, with a mortality rate of 4.6 per 100,000 children at risk (13). The advent of novel targeted therapies has escalated the risk of TLS, underscoring the need for robust preventive measures (3). Therefore, this study aimed to evaluate the relationship between dynamic biochemical changes and AKI severity in Egyptian children with TLS, using a nephrological framework based on pRIFLE criteria (14).

## Patients and methods

This retrospective study included all children (<18 years) with hematologic malignancies who developed TLS at Borg El-Arab Pediatric Oncology Center between January 2022 and June 2025. The study was conducted in accordance with the principles of the Declaration of Helsinki. Ethics approval was obtained from the Ethics Committee (IRB Number 00012098), Faculty of Medicine, Alexandria University, Egypt. Informed ascent/consent was obtained from all the patients/legal guardians before the study. Patient data were collected from medical records, and confidentiality was maintained. TLS was classified according to the Howard–Pui modification of the Cairo–Bishop criteria into two “traditional” subtypes: laboratory TLS (LTLS) and clinical TLS (CTLS) (1, 15). Based on the pRIFLE criteria (eGFR and urine output) (14), patients were further stratified into “nephrological” or “AKI severity” subgroups, including mild AKI (pRIFLE-0/R/I) and severe AKI (pRIFLE-F). In this study, eGFR was calculated using the modified Schwartz 2009 equation (16).

Accordingly, patients within traditional and nephrological subgroups were compared as regards: demographic data, pre-existing renal conditions, nephrotoxic drug burden (calculated retrospectively according to Ehrmann et al. (17) for the 5 days preceding TLS onset), and anthropometric measurements. Likewise, comparisons highlighting tumor-disease-burden were carried out, including: the type of hematological malignancy, white blood cell (WBC) count in leukemia, lactate dehydrogenase (LDH) level, tumor bulk, lymphadenopathy, and organomegaly. Radiological findings, particularly kidney infiltration, renal vein thrombosis, malignant effusion, and mediastinal masses, were also analyzed.

Studied parameters of TLS included: onset (spontaneous or treatment-induced), duration, clinical manifestations (such as tetany, arrhythmia, or oliguria), and outcome. TLS risk groups in chemotherapy-induced cases were defined according to the consensus proposed by Cairo and the TLS Expert Panel (18). Both TLS prophylactic measures undertaken in treatment-induced cases (hydration and hypouricemic agents) and definitive treatment (such as hydration, phosphate-binding and hypouricemic agents, anti-hyperkalemic measures, diuretics, and dialysis) were reported. We analyzed the trends of 10-day (3 days before and 7 days after initiation of cytotoxic chemotherapy) serial laboratory measurements of blood counts, urea, creatinine, eGFR, albumin, sodium, potassium, calcium, phosphorus, and uric acid. The interpretation of serum phosphate levels was based on age-specific normal serum phosphate ranges (19).

Data was analyzed using IBM SPSS Statistics, version 20.0 (IBM Corp., Armonk, NY, USA). Categorial variables are presented as numbers (n) and percentages (%), while continuous variables are presented using range (minimum and maximum), median, and interquartile range (IQR). Chi-square, Fisher’s Exact, or Monte Carlo correction, Student-t, and Mann-Whitney tests were used in the univariate analyses to compare subgroups, exact tests, considering the small sample size and nonnormality of the variables. Significance of the obtained results was judged at the 5% level. The ROC curve was used to compare the performance of delta phosphate (ΔPh) and delta uric acid (ΔUA) as predictors of AKI severity before TLS onset and to check the cutoff values. During the preparation of this work, the authors used ChatGPT to assist with language editing. After using this tool, the authors reviewed and edited the content as needed and take full responsibility for the content of the publication.

## Results

In this retrospective TLS cohort of 50 children (males, 66%; median age, 93 months) with hematological malignancies (54% leukemia, predominantly T-ALL; 46% lymphoma, mainly Burkitt’s lymphoma), most patients had CTLS (86%). At presentation, 12 (66.7%) of the 18 patients with T-ALL had a mediastinal mass, and 10 (55.6%) had a WBC count >100 x 10^9^/L. Neither finding was observed in any of the 7 B-ALL patients, as shown in **Table 1**: demographic data.

**Table 1:** Background characteristics of patients and tumor burden.

Demographic and clinical data	All patients (n=50)	Traditional TLS Subgroups			Nephrological Subgroups		
		Laboratory TLS (n=7)	Clinical TLS (n=43)	*p*	Mild (pRIFLE-0/R/I) (n=20)	Severe (pRIFLE-F) (n=30)	*P*
Age in months							
Range	14 – 182	33-182	14-174	0.873	14.0-182.0	24.0-168	0.729
Median, IQR	93 (54 – 132)	86 (52.5–120.5)	93 (56.5– 130.5)		84.5 (52.0– 131.0)	94 (54 – 132)	
Age >10 years, n (%)	20 (40%)	2 (28.6%)	18 (41.9%)	0.681	8 (40%)	12 (40%)	1.00
Males, n (%)	33 (66%)	3 (42.9)	30 (69.8)	0.210	13 (65%)	20 (66.7)	0.903
Underlying malignancy							
Leukemia	27 (54%)	3 (42.9%)	24 (55.8%)		10 (50%)	17 (56.7)	
B-ALL	7 (25.9%)	2 (66.7%)	5 (20.8%)	0.156	2 (20%)	5 (29.4%)	0.678
T-ALL.	18 (66.7%)	1 (33.3%)	17 (70.8%)	0.250	6 (60%)	12 (70.6%)	0.683
AML	2 (7.4%)	0	2 (8.3%)	1.000	2 (20%)	0	0.128
Lymphoma	23 (46%)	4 (57.1%)	19 (44.2%)	1.000	10 (50%)	13 (43.3%)	0.229
Burkitt's lymphoma	20 (87%)	4 (100%)	16 (84.2%)		10 (50%)	10 (76.9%)	
Burkitt's leukemia	3 (13%)	0	0		0	3 (23.1%)	
Nephrotoxic drug burden^#^	1 (2%)	0	1 (2.3%)	1.000	1	0	4.00
Tumor burden							
LDH (U/L), median (IQR)	(n=21)	(n=3)	(n=18)		(n=8)	(n=13)	
	2315.0 (1520.0 – 2920.0)	1460.0 (1199.5 – 1901.0)	2367.5 (1620.0 – 3420.0)	0.221	2169.0 (1390.0 – 2381.0)	2570 (1620 – 3420)	0.500
LDH >2 ULN	18 (85.7%)	2 (66.7%)	16 (88.9%)	1.000	7 (87.5%)	11 (84.6%)	0.853
WBC count (x 10^9^/L)							
Range	5.3 – 550	5.8 – 49	5.3 – 550	0.149	5.8 – 413	5.3 – 550	0.905
Median (IQR)	29.1 (12.9–87.7)	15.1 (13.3–27.45)	31.3 (13.15–110)		16.8 (13.05–160.9)	30.65 (12.9 – 77)	
Hyperleukocytosis (WBCs >100x10^9^/L)	(n=27)	(n=3)	(n=24)		(n=10)	(n=17)	
	12 (44.4%)	0	12 (50%)	0.065	6 (60%)	6 (35.3%)	0.257
Bulky tumor (>10 cm)	14 (28%)	0	14 (32.6%)	0.169	4 (20%)	10 (33%)	0.304
Organomegaly	46 (92%)	6 (85.7%)	40 (93%)	0.464	18 (90%)	28 (93.3%)	1.000
Lymphadenopathy	45 (90%)	7 (100%)	38 (88.4%)	0.904	18 (90%)	27 (90%)	1.000
Radiological findings at admission^$^							
Mediastinal mass	14 (28%)	–	14 (32.6%)	–	4 (20%)	10 (33.3%)	0.304
Renal infiltration	10 (20%)	–	10 (23.3%)	–	3 (15%)	7 (23.3%)	0.720
Pleural effusion	12 (24%)	–	12 (27.9%)	–	2 (10%)	10 (33.3%)	0.091
Pericardial effusion	2 (4%)	–	2 (4.7%)	–	0	2 (6.7%)	0.511

# Calculated in the 5 days preceding TLS onset, and only one patient had received a nephrotoxic drug (gentamycin).

$ None of the patients had renal vein thrombosis at admission (not shown in **Table 2**).

AML, acute myeloid leukemia; ULN, upper limit of normal; WBC, white blood cells.

LDH: lactate dehydrogenase.

ALL: acute kymphoblastic leuekemia.

pRIFLE: Pediatric Risk, Injury, Failure, Loss, End-stage renal disease.

TLS: Tumor Lysis Syndrome.

*: Statistically significant at p ≤ 0.05.

On admission, data on risk assessment were complete for only 16 (of 22) patients with chemotherapy-induced TLS, and all were classified as high-risk for TLS none of the patients had a pre-existing renal condition. They uniformly received hydration and febuxostat due to our limited access to rasburicase and the high risk of glucose 6-phosphate dehydrogenase (G6PD) deficiency in our population, as a prophylaxis (not shown in **Table 2**). Of the Howard-Pui laboratory criteria, hyperkalemia was the least frequently encountered, in 7 patients (14%), and hyperphosphatemia was the most common (94%).

**Table 2:** Characteristics and outcome of TLS attacks.

TLS characteristics	All patients (n=50)	Traditional TLS Subgroups			Nephrological Subgroups		
		Laboratory TLS (n=7)	Clinical TLS (n=43)	*p*	Mild (pRIFLE-0/R/I) (n=20)	Severe (pRIFLE-F) (n=30)	*p*
TLS onset							
Spontaneous	28 (56%)	3 (42.9%)	25 (58.1%)	0.684	6 (30%)	22 (73.3%)	0.002*
Chemotherapy-induced	22 (44%)	4 (57.1%)	18 (41.9)		14 (70%)	8 (26.7%)	
TLS duration (days)							
Range	2 – 11	2 – 3	2 – 11	<0.001^*^	2 – 6	2 – 11	<0.001^*^
Median (IQR)	5 (3 – 7)	2 (2 – 3)	5 (3 – 7)		3 (2 – 3)	6.5 (5 – 8)	
TLS Howard-Pui laboratory criteria, n (%)							
Phosphorus≥ 6.5 mg/dL or 25% ↑ above baseline	47 (94%)	6 (85.7%)	41 (95.3%)	0.370	18 (90%)	29 (96.7%)	0.556
Uric acid>ULN for age or 25% ↑ above baseline	41 (82%)	5 (71.4%)	36 (83.7%)	0.595	13 (65%)	28 (93.3%)	0.021*
Potassium≥6 mmol/L or 25% ↑ above baseline	7 (14%)	0	7 (16.3%)	0.573	2 (10%)	5 (16.7%)	0.687
Corrected calcium≤7 mg/dL, or ionized calcium level <4.5 mg/dl or or 25% ↓ below baseline	45 (90%)	5 (71.4%)	40 (93%)	0.138	17 (85%)	28 (93.3%)	0.377
TLS Howard-Pui clinical criteria, n (%)							
Neurologic: etany or seizures	9 (18%)	–	9 (20.9%)	0.325	1 (5%)	8 (26.7%)	0.067
Cardiac: arrhythmia or sudden death	5 (10%)	–	5 (11.6%)	1.000	3 (15%)	2 (6.7%)	0.377
AKI: Creatinine above 1.5 UNL or 0.3 ↑ above baseline	41 (82%)	–	41 (95.3%)	<0.001^*^	11 (55%)	30 (100%)	<0.001^*^
AKI: Oliguria (<0.5 ml/hour)>6 hours	17 (34%)	–	17 (39.5%)	0.080	1 (5%)	16 (53.3%)	<0.001^*^
Nephrological subgroups, n (%)							
PRIFLE-0/R/I (Mild)	20 (40%)	7 (100%)	13 (30.2%)	<0.001^*^	20 (40%)	0	–
0: No AKI	4 (8%)	3 (42.9%)	1 (2.3%)		4 (20%)	0	
R: Risk	6 (12%)	4 (57.1%)	2 (4.7%)		6 (30%)	0	
I: Injury	10 (20%)	0	10 (23.3%)		10 (50%)	0	
PRIFLE-F (Severe)	30 (60%)	0	30 (69.8%)		0	30 (100%)	
Treatment, n (%)							
Hypouricemic agents (Febuxostat)#	49 (98%)	7 (100%)	42 (97.7%)	–	20 (100%)	29 (96.7%)	–
Phosphate binder (sevelamer)	46 (92%)	6 (85.7%)	40 (93%)	0.464	18 (90%)	28 (93.3%)	1.000
Anti-hyperkalemic measures	7 (14%)	0	7 (16.3%)	0.573	2 (10%)	5 (16.7%)	0.687
Diuretics	34 (68%)	1 (14.3%)	33 (76.7%)	0.003^*^	8 (40%)	26 (86.7%)	0.003^*^
Kidney replacement therapy	31 (62%)	0	31 (72.1%)	0.001^*^	4 (20%)	27 (90%)	0.001^*^
Pediatric intensive care admission	33 (66%)	0	33(76.7%)	<0.001^*^	9 (45%)	24(80%)	0.010*
Mechanical ventilation	20 (40%)	0	20 (46.5%)	0.033*	4 (20%)	16 (53.3%)	0.018*
TLS outcome							
Resolved	44 (88%)	7 (100%)	37 (86%)	0.576	20 (100%)	24 (80%)	0.069
Died	6 (12%)	0	6 (14%)		0	6 (20%)	

# Only one patient with CTLS and severe (pRIFLE-F) AKI had received allopurinol as a treatment for hyperuricemia.

AKI: Acute Kidney Injury. pRIFLE: Pediatric Risk, Injury, Failure, Loss, End-stage renal disease. pRIFLE-0: No Kidney Injury.

pRIFLE-R: Risk of kidney dysfunction. pRIFLE-I: Injury to the kidney. pRIFLE-F: Failure of kidney function. TLS: Tumor Lysis Syndrome.

ULN: Upper Limit of Normal.

IQR: Interquartile Range.

*: statistically significant difference (p < 0.05).

↑ / ↓: Increase or decrease from baseline.

Demographic characteristics, nutritional status (z-scores of weight and height, not shown in **Table 2**), malignancy type, tumor burden, and radiological findings (including kidney infiltration) were comparable between patients with mild TLS-related AKI (pRIFLE-0/R/I, 40%) and their severe counterparts (pRIFLE-F, 60%), as shown in **Table 2**. In contrast, pRIFLE-F was strongly associated with CTLS and spontaneous TLS onset, prolonged TLS duration (median 6.5 days vs 3 days for mild AKI), increased need for KRT, intensive care admission, and higher mortality.

On admission, except for uric acid and creatinine levels that were higher in the severe AKI group, most static laboratory markers (phosphorus, calcium, potassium, and urea) were similar in mild and severe AKI TLS subgroups, as shown in **Table 3**. This also applies to clinical examination (blood pressure, heart rate, and temperature), albumin, sodium, coagulation profile, hemoglobin, white blood cells, and platelet counts (not shown in **Table 3**). Upon TLS evolution, and as predicted, patients with severe AKI had higher peak urea, creatinine, uric acid, and phosphate and lower nadir eGFR. However, unlike uric acid, the percent rise in phosphate was significantly greater in the severe AKI subgroup.

**Table 3:** Biochemical markers of TLS and their trend kinetics.

Biochemical markers	All patients (n=50)	Traditional Subgroups			Nephrological Subgroups		
		Laboratory TLS (n=7)	Clinical TLS (n=43)	*p*	Mild (pRIFLE-0/R/I) (n=20)	Severe (pRIFLE-F) (n=30)	*p*
Phosphate kinetics (mg/dL)							
Phosphorus at admission							
Range	1.6 – 17	3.2 – 8.3	1.6 – 17	0.787	2.1 – 8.3	1.6 – 17	0.618
Median (IQR)	4.7 (3.4 – 5.5)	5.2 (4.3– 5.3)	4.6 (3.4 – 5.6)		4.65 (3.5 – 5.55)	4.70 (3.4 – 5.5)	
Hypophosphatemia at presentation	15 (30%)	1 (14.3%)	14 (32.6%)	0.420	6 (30%)	9 (30%)	1.0
Hypophosphatemia before TLS onset in treatment-induced TLS	(n=22)	(n=4)	(n=18)		(n=14)	(n=8)	
	9 (40.9%)	1 (25%)	8 (44.4%)	0.660	5 (37.7%)	4 (50%)	1.0
Peak phosphorus level, median (IQR)	12.1 (9.45 –16.9)	8.55 (8 – 8.9)	12.7(10.1 –17.9)	<0.001^*^	9.35 (8.3 –10.9)	15 (12.1 –20)	0.001^*^
Phosphorus percent change from admission to the peak, median (IQR)	160.2 (78.46 – 309.7)	53.85 (43.78 – 78.36)	205.5 (88.22 – 350.2)	<0.001^*^	92.11 (54.62 – 195.7)	271.3 (116 – 380)	0.002^*^
Phosphorus at lowest eGFR, median (IQR)	8.65 (7.2–11.1)	7.5 (6.35 – 8.55)	8.7 (7.25–11.3)	0.138	7.85 (6.35- 8.80)	9.25 (7.80–12.50)	0.041^*^
Phosphate total change/day							
Range	0.25 – 8.5	1.15 – 1.85	0.25 – 8.5	0.300	0.25 – 5.1	1.2 – 8.5	0.008*
Median (IQR)	2.05 (1.15 – 3.65)	1.55 (1.28 – 1.78)	2.5 (1.13 – 3.7)		1.55 (1.07 – 2.4)	3.68 (2.4 – 4.75)	
Uric acid kinetics (mg/dL)							
Uric acid at admission							
Range	2.4 – 50	5.3 – 15.8	2.4 – 50	0.661	2.4 – 15.8	3.1 – 50	0.002*
Median (IQR)	8.65 (5.4 – 13.2)	7.30 (5.45– 11.05)	9.2 (5.95 – 13.25)		6.0 (5.25 – 10.15)	10.85 (7.5 – 16)	
Peak uric acid							
Range	2.4 – 50	5.5 – 15.8	2.4 – 5	0.476	2.40 – 27.40	7 – 50	0.006^*^
Median (IQR)	12.4 (8.8 – 16)	11 (8.55 – 14.45)	13 (8.9 – 16.7)		9.9(6.25 – 12.95)	13.3 (10.5 – 18)	
Uric acid total change/day							
Range	-3.40 – 10.4	-3.4 – 8.7	-2.35 – 10.4	0.967	-3.40 – 10.4	-2.35 – 6.4	0.110
Median (IQR)	0.0 (-0.9 – 2.2)	0.75 (-1.7 – 5.1)	0.0 (-0.9 – 2.2)		0.85 (0.0 – 2.2)	-0.7(-1.22 – 2.75)	
Potassium kinetics (mmol/L)							
Potassium at admission							
Range	3– 7.8	3.2 – 5.2	3 – 7.8	0.436	3 – 5.5	3 – 7.8	0.637
Median (IQR)	4.1 (3.6 – 4.6)	4.7 (3.95 – 4.95)	4.1 (3.55 – 4.5)		4.1 (3.5 – 4.7)	4.15 (3.6 – 4.5)	
Hypokalemia at presentation	8 (16%)	1 (14.3%)	7 (16.3%)	0.893	4 (20%)	4 (13.3%)	0.532
Hypokalemia before TLS onset in treatment-induced TLS	(n=22)	(n=4)	(n=18)		(n=14)	(n=8)	
	4 (18.2%)	0	4 (22.2%)	0.418	3 (18.4%)	1 (12.5%)	1.00
Potassium at TLS onset							
Range	3.1 – 7.8	3.2 – 5.3	3.1 – 7.8	0.308	3.2 – 6.2	3.1 – 7.8	0.410
Median (IQR)	4.3 (3.8 – 4.7)	4.6 (4.25 – 4.85)	4.3 (3.8 – 4.55)		4.45 (3.9 – 4.8)	4.25 (3.8 – 4.6)	
Potassium at the lowest eGFR							
Range	2.9 – 9.1	3.2 – 5	2.9 – 9.1	0.394	2.9 – 6.5	3.4 – 9.1	0.416
Median (IQR)	4.5 (3.9 – 5)	4.3 (3.9 – 4.75)	4.5 (3.9– 5.1)		4.4 (3.9– 4.9)	4.5 (4 – 5.2)	
Calcium at admission (mg/dl)							
Range	3.9 – 13	7.7 – 9.9	3.9 – 13	0.772	7.1 – 9.9	3.9 – 13	0.836
Median (IQR)	8.5 (7.7 – 9)	8.4 (8.3 – 8.7)	8.5 (7.7 – 9.05)		8.50 (8.1 – 9.05)	8.45 (7.7 – 9)	
Creatinine at admission (mg/dL)							
Range	0.2 – 5.64	0.34 – 0.85	0.2 – 5.64	0.193	0.2 – 1.2	0.27 – 5.64	0.022^*^
Median (IQR)	0.64 (0.47 – 1.05)	0.49 (0.47 – 0.47)	0.66 (0.47 – 0.47)		0.58 (0.43 – 0.7)	0.85 (0.51 – 2.20)	
Urea kinetics (mg/dL)							
Urea at admission							
Range	9 – 285	11 – 47	9 – 285	0.722	9 – 67	9 – 285	0.159
Median (IQR)	26 (15– 56)	41 (15 – 45)	26 (15 – 59)		25 (15 – 43)	26 (17 – 60)	
Urea at the onset of TLS							
Range	9 – 285	17 – 79	9 – 285	0.510	11 – 120	9 – 285	0.714
Median (IQR)	56 (26 – 86)	51 (42 – 55)	60 (26 – 97.5)		52.5 (36.5–76)	60 (26–111)	
Urea at the lowest eGFR							
Range	17 – 323	17 – 84	26 – 323	<0.001^*^	17 – 118	47 – 323	<0.001^*^
Median (IQR)	94 (62– 175)	47 (37.5–68.5)	113 (80–180)		79 (46 – 90)	149 (94–201)	

eGFR: Estimated Glomerular Filtration Rate

IQR: Interquartile Range

pRIFLE: Pediatric Risk, Injury, Failure, Loss, End-stage renal disease criteria.

TLS: Tumor Lysis Syndrome.

*: statistically significant difference (p < 0.05).

**Figure 1:**
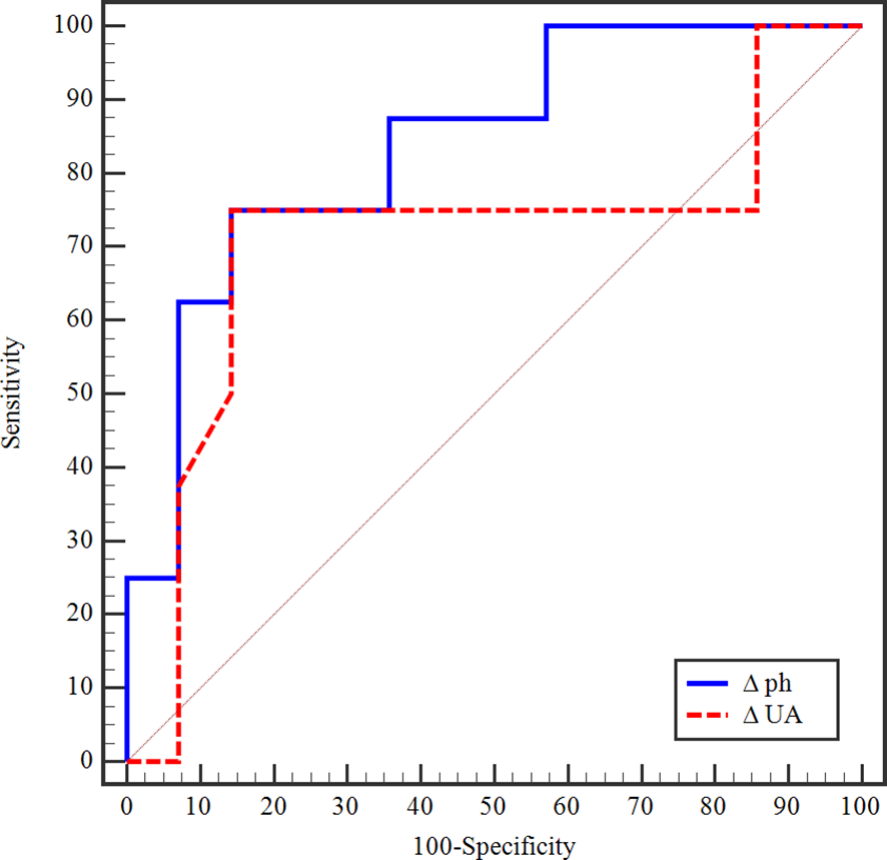
ROC curve for delta phosphorus and uric acid to predict severity of AKI in TLS (severe vs mild pRIFLE stages).

The daily change in serum phosphate level (ΔPh) before TLS onset in chemotherapy-induced TLS (n=22) was a strong discriminator of AKI severity. As demonstrated by the ROC curve (**Figure 1**), Δ Ph yielded an AUC of 0.839 (p=0.009), indicating good predictive accuracy for identifying severe pRIFLE-F cases. A cutoff value of >2.6 mg/dL provided 75% sensitivity and 85.7% specificity. In contrast, the predictive performance of the change in uric acid (ΔUA) was modest, with an AUC of 0.710 (p=0.109). Using a cutoff of ≤0.5 mg/dL, Δ UA showed 62.5% sensitivity and 85.7% specificity, but did not reach statistical significance as shown in **Table 4**. Notably, 9 patients with chemotherapy-induced TLS (of 22, 40.9%) were hypophosphatemic before the onset of TLS.

**Table 4:** Prognostic performance for delta phosphorus and uric acid to predict severity of AKI in TLS (severe vs mild pRIFLE stages).

	AUC	*p*	95% CI	Cut off^#^	Sensitivity	Specificity	PPV	NPV
Δ phosphorus	0.839	0.009^*^	0.665 – 1.013	>2.6^#^	75.0	85.71	75.0	85.7
Δ uric acid	0.710	0.109	0.448 – 0.971	≤-0.5	62.50	85.71	71.4	80.0

AUC, Area Under a Curve; CI, Confidence Intervals; NPV, Negative predictive value; PPV, Positive predictive value.

*: Statistically significant at p ≤ 0.05.

#Cut off was chosen according to the Youden index.

With the high burden of spontaneous (56%) and severe-AKI TLS (60%), 31 patients (62%) underwent conventional hemodialysis. Daily dialysis sessions were required for a median duration of 3 days (range1-9). Six patients (12%) died before the TLS attack resolved. Only 1 patient died due to volume overload and pulmonary edema. Death in the 5 other patients was due to the development of nosocomial infection after the onset of TLS, complicated by septic shock and multiorgan failure. This hindered the resumption of dialysis, which was required to correct the severe metabolic derangement in 3 of them and the volume overload in the other two; continuous renal replacement therapy (CRRT) was not available for the patients who survived, none had residual renal impairment at 3 months post-TLS.

## Discussion

In this retrospective cohort of children with hematological malignancies who developed TLS, AKI severity emerged as the principal determinant of clinical outcome. Using a nephrological framework based on pRIFLE criteria, severe AKI (pRIFLE-F) was strongly associated with CTLS, spontaneous TLS onset, prolonged TLS duration, increased need for KRT, intensive care admission, and higher mortality.

Spontaneous TLS was observed frequently in our cohort (56%), implying delayed presentation of patients to the health facility and more advanced cancer stages at diagnosis, obviating the chance for TLS prophylaxis. Generally, spontaneous TLS is more common in pediatric patients (30-72%) (7, 9, 12, 20) than in adults (~20%) (21, 22). In this study, it occurred significantly more often in children with severe AKI. Similar observations have been reported in pediatric series from areas with resource constraints, where delayed presentation and high tumor burden at diagnosis contribute to severe renal complications (10, 12). This highlights the importance of early risk stratification at diagnosis, particularly in settings with limited access to rasburicase and early dialysis.

Another key feature of this study is the high incidence of CTLS (86%), similar to that reported in a recent pediatric study (80%) (12) but contradicting the 3-6% incidence in earlier adult reports (23–25). Inherent to their definitions, CLTS was paralleled by a 60% incidence of severe AKI. This supports pediatric data indicating that CTLS reflects a higher metabolic burden with direct renal consequences (15, 26). In contrast, some adult studies reported severe AKI in LTLS. However, they classified CTLS based on clinical manifestations and excluded creatinine rise as a criterion (24).

Among the laboratory criteria for TLS, hyperuricemia was observed in 82% of patients with a median peak uric acid level of 12.4 mg/dL (range 2.4-50). The selection of a hypouricemic agent is guided by the patient’s TLS risk profile, the urgency of cytoreduction, and potential drug toxicities (3). The British Society for Haematology (BSH) recommends xanthine oxidase (XO) inhibitors, such as allopurinol, for patients with low or intermediate risk of TLS (27). However, for pediatric patients with high-risk tumors, like the highly proliferative treatment-sensitive hematological malignancies in the present cohort, guidelines advise against delaying chemotherapy while awaiting XO inhibitors to reduce uric acid production (28–30). In such high-risk scenarios, rasburicase, a recombinant urate oxidase, facilitates the rapid oxidation of uric acid into the more soluble allantoin, typically decreasing serum uric acid to 1 mg/dL within 4 hours (31). This rapid decline has been demonstrated to improve kidney function in randomized trials in children (32, 33).

Nevertheless, the benefits of rasburicase in reducing uric acid levels and preventing AKI are not universally accepted, as several multivariate modeling studies suggest that rasburicase may not significantly alter the risk of TLS-related AKI (34). Early use of rasburicase in the course of AKI may mitigate further kidney damage, but does not significantly improve the outcome of severe AKI (34, 35). Furthermore, Rasburicase carries a risk of severe adverse reactions, including hemolysis and methemoglobinemia (36), hence the BSH recommends testing for G6PD deficiency before starting it in risky ethnicities, including the Mediterranean region (27). Despite being the established cornerstone of TLS prophylaxis for high-risk patients (3, 27), none of our patients with non-spontaneous TLS received rasburicase. This was primarily due to the unavailability of the drug at our hospital and due to institutional resource constraints, a challenge compounded by the high risk of G6PD deficiency in the Egyptian population.

In contrast, nearly all our cohort received febuxostat for prophylaxis or treatment of TLS. Febuxostat, a non-purine XO inhibitor, has greater selectivity and offers several pharmacological advantages over allopurinol; it does not require dose adjustments in patients with mild or moderate renal impairment, and has fewer interactions (37–39). In an adult randomized clinical trial, a single fixed dose of febuxostat achieved significantly superior uric acid control compared to allopurinol, while maintaining comparable safety profiles and renal preservation (37). Although data in the pediatric population remain limited, a retrospective study of 45 children demonstrated similar efficacy between the two agents (39). Despite these advantages, febuxostat remains less popular than allopurinol, due to its higher cost (40), and concerns regarding a potential association with higher all-cause and cardiovascular mortality in long-term studies (41). Currently, febuxostat is approved for adults with TLS in the European Union, the USA, and Japan (3), but its safety and efficacy in children (<18 years) have not been established yet, and the 2025 BSH guidelines do not recommend it (27). In our setting, however, the logistic constraints and the practical benefits of febuxostat justified its use as a vital component of TLS management.

In low- and middle-income countries where access to rasburicase is limited, the use of XO inhibitors is a pragmatic, albeit problematic, choice. XO inhibitors reduce uric acid synthesis at the expense of xanthine accumulation. This can be counterproductive as xanthine is less soluble than uric acid and can lead to “xanthine nephropathy”, potentially exacerbating AKI despite well-controlled serum uric acid levels (42, 43). This complication is frequently overlooked as xanthine levels are rarely measured in clinical practice (44). Howard et al. (3), in their recent review on TLS, raised the question ‘Are XO inhibitors better than nothing?’ highlighting the urgent need for randomized trials comparing best supportive care with and without XO inhibitors in rasburicase-scarce settings. However, the authors acknowledged that such a trial would be ethically controversial, as XO inhibitors have been used for almost a century with good outcomes, therefore withholding them would be viewed as unethical. On the other hand, continuing to prescribe agents that have never definitively been shown to reduce TLS or AKI with a risk of xanthine nephropathy is equally questionable.

Amidst this controversy, the current study provides important real-world data. In our cohort of 50 pediatric patients, classified as high-risk for TLS, spontaneous TLS and CTLS were observed in 56% and 86% of patients, respectively. Despite the universal use of XO inhibitors, 46 patients (92.9%) developed AKI, which reached the most severe stage (pRIFLE-F) in 30 (60%), and 31 children (62%) required KRT. These outcomes contrast with the 32% and 29% incidence of pRIFLE-F and KRT, respectively, in a comparable pediatric TLS cohort (n=31), with rasburicase used in 50% of their patients (12) and the 9% rate of KRT with the more widespread use of rasburicase (45). Accordingly, universal access to rasburicase is critical, and every effort to reduce disparities in access to essential medications for TLS management should be exerted (3).

Aligning with other pediatric studies (12, 45), hyperkalemia was uncommon and occurred at similar frequencies across AKI groups, while hyperphosphatemia was the most frequent TLS-laboratory criterion. Hypocalcemia was nearly universal and reflected phosphate elevation rather than renal injury severity (1, 3, 15). The mechanism of hyperphosphatemia in TLS is controversial, but it is currently viewed as a consequence of kidney injury and decreased renal excretion of phosphate (12).

Interestingly, we observed hypophosphatemia preceding the onset of chemotherapy-induced TLS in 9 patients (40.9%) and hypokalemia in 4 (18.2%), but there was no significant difference in the presence of pre-TLS hypophosphatemia among AKI severity groups. The most plausible explanation for hypophosphatemia is increased phosphate utilization by malignant cells as the tumor progresses (46). ‘Tumor genesis syndrome’ (TGS) is the opposite, yet closely related, condition to TLS caused by neoplastic cytogenesis, most frequently observed in malignancies with high cell burden. Hypophosphatemia is the most common electrolyte abnormality associated with TGS; however, hypokalemia can also occur (47, 48). Biró et al. (12) reported hypophosphatemia before the introduction of chemotherapy in 61% of their patients, identifying it as a significant risk factor for TLS. They hypothesized that depletion of extracellular and, consequently, intracellular phosphate may decrease intracellular ATP production, impair tubular phosphate reabsorption, and increase its excretion, leading to greater calcium phosphate deposition. These pathological events would eventually lead to severe TLS and promote TLS-related AKI (12, 49). Moreover, Biró et al. (12) did not find any significant defect of tubular phosphate excretion in patients without AKI (12).

In this study, neither demographic data nor tumor type were statistically associated with AKI severity. In contrast, Biró et al (12) observed pRIFLE-F to be associated with older age in children with TLS, and Anderson et al (45) found that 18 of 20 children who required CRRT for TLS were males. In another cohort, 87.8% of children who required dialysis were male (50).

Surprisingly, laboratory surrogates of tumor burden (WBC count, LDH), organomegaly, lymphadenopathy, and radiological findings, including renal infiltration and mediastinal masses, were not statistically correlated with AKI severity. Although baseline uric acid and creatinine levels were higher in the severe AKI group, phosphorus levels were similar in the mild and severe AKI TLS subgroups. Similarly, serum uric acid, phosphorus, and LDH levels in patients with no/mild AKI were comparable to those in patients with severe AKI in a pediatric cohort of 20 patients who underwent CRRT for TLS (45).

One would expect a higher tumor burden to reflect a greater metabolic load, resulting in increased uric acid and calcium-phosphate crystal formation, and therefore more severe kidney involvement. However, the lack of a significant difference in tumor burden and phosphate levels across AKI severity groups in the present study may be explained by emerging crystal-independent mechanisms of TLS-related AKI (4). Historically, reports of renal calcium-phosphate deposition in renal tubules date back to the 1970s, when alkalinization was widely practised, thereby favouring phosphate deposition during hyperphosphatemia, which is rarely observed nowadays due to the early implementation of KRT (51). In modern practice, the use of rasburicase has become the standard of care for TLS prophylaxis in high-risk patients, but many still experience AKI without any evidence of hyperphosphatemia or hyperuricemia (4). Studies have described similar rates of TLS-related AKI (~30%) in both the rasburicase era (52) and earlier, when allopurinol and alkalinization were primarily used (24). Consequently, Arnaud et al. (4) explored the pathomechanisms of crystal-independent TLS-related AKI *in vitro* and in a murine *in vivo* model, identifying extracellular histones released in large amounts during TLS that profoundly damage the endothelium. Finally, it is worth noting that 56% of our patients presented with spontaneous TLS, and the remaining patients had a high TLS risk. This uniform high tumor burden across our cohort may have created a ceiling effect, masking potential correlations between tumor burden and AKI severity in our analysis.

Baseline and follow-up levels of other electrolytes, including potassium, calcium, and sodium, did not discriminate AKI severity. Likewise, serum albumin, hemoglobin, platelet counts, and coagulation parameters were not associated with AKI severity, confirming their limited prognostic value in pediatric TLS-induced AKI (1, 9, 12).

Among the biochemical parameters observed during the evolution of chemotherapy-induced TLS, phosphate kinetics emerged as the strongest predictor of AKI severity. Although hyperphosphatemia was nearly universal among patients with TLS, those who progressed to severe AKI had significantly higher median peak phosphate levels (15 vs 9.35 mg/dL), a greater percent increase (271.3 vs 92.11%), and a markedly higher daily rise in serum phosphate (3.68 vs 1.55 mg/dL) before AKI onset. Our analysis showed that ΔPh had strong discriminatory performance for predicting severe AKI (AUC = 0.839, p = 0.009), with a cutoff of >2.6 mg/dL, 75% sensitivity, and 85.7% specificity. These findings align with mechanistic and clinical data indicating that phosphate accumulation is an early and strong indicator of TLS-related AKI (11, 12, 15, 45, 52).

In adults, Darmon et al. (52) reported that a 1 mmol/L (3.1 mg/dL) increase in serum phosphate level was associated with a five-fold increase in CTLS risk, while Lemerle et al. (11) reported the warning peak value of serum phosphate was 2.1 mmol/L (6.5 mg/dL). Similarly, Biró et al. (12) reported a significant discriminatory capacity of daily ΔPh for severe TLS–AKI in pediatrics (cutoff 0.32 mmol/L). Anderson et al (45) observed that children with severe AKI had significantly higher serum phosphate levels before initiation of CRRT (6.4 mg/dL with no/mild AKI vs.10.5 mg/dL with severe AKI), and the phosphate level at 18 hours before CRRT was the best predictor of severe AKI. They recommended that a rapid rise in phosphate levels should signify consideration of CRRT to prevent the development or progression of AKI. Abdel-Nabey et al. (53) initiated KRT in adult patients with TLS admitted to the intensive care unit at a phosphate level of >7.7 mg/dL or when the level increased by >3 mg/dL every 6 hours. In conclusion, due to significant age-related variation in normal phosphate levels in children and the frequently reported initial hypophosphatemia, it may be preferable to adopt an approach based on dynamic changes in serum phosphate levels rather than fixed thresholds.

In contrast, uric acid dynamics demonstrated a different pattern. Absolute uric acid levels at admission and peak values were significantly higher in children with severe AKI, supporting its pathogenic role in TLS-related kidney injury (52). However, early changes in uric acid (ΔUA) did not predict AKI severity, consistent with recent pediatric data indicating that uric acid kinetics are less informative than phosphate dynamics for early risk stratification (12). This differs from an adult study, where uric acid was the most sensitive predictor according to the ROC curve (5).

Severe AKI was associated with substantial clinical consequences; 31 patients (62%) underwent hemodialysis, and six (12%) passed away before the resolution of the TLS. There is no consensus on the modality and timing of KRT in TLS (54). Early introduction of KRT has been associated with better long-term kidney survival (54, 55), but with the risk of increased rates of dialysis catheter-related infections (54, 56). CRRT, if available, would have been the standard of care type of dialysis for the 5 patients in whom septic shock and multiorgan failure hindered the continuation of conventional hemodialysis (45). Collectively, patients with pRIFLE-F were significantly more likely to require pediatric intensive care admission and mechanical ventilation, with higher TLS-related mortality. These findings are consistent with previous studies demonstrating that TLS-associated AKI is a major driver of morbidity, dialysis requirement, and mortality in hematologic malignancies (15, 57–59).

Despite the severity of the acute course, renal outcomes among survivors were favorable. All surviving children demonstrated complete renal recovery at three-month follow-up, consistent with prior pediatric studies showing that TLS-associated AKI is typically transient when metabolic abnormalities are promptly corrected (3).

This study has several limitations. Its retrospective, single-center design and modest sample size limit generalizability. The high incidence of spontaneous TLS and the uniformity of prophylactic approaches precluded evaluation of preventive strategies as outcome modifiers. Additionally, incomplete long-term biochemical follow-up in mild cases limited late comparisons. Nevertheless, the consistency of our findings with emerging pediatric TLS literature strengthens their validity.

In conclusion, this study demonstrates that in pediatric TLS, AKI severity, rather than malignancy type or static laboratory abnormalities, determines clinical outcomes. Dynamic phosphate changes, particularly rapid rises in serum phosphate, represent a robust early marker of severe AKI and may offer a practical tool for early risk stratification, especially in resource-limited settings.

## Data Availability

The original contributions presented in the study are included in the article/supplementary material. Further inquiries can be directed to the corresponding author.
